# The Effects of Parental Emotion Regulation Ability on Parenting Self-Efficacy and Child Diet

**DOI:** 10.1007/s10826-020-01745-x

**Published:** 2020-06-24

**Authors:** Sara J. Sagui-Henson, Laura Marie Armstrong, Alexis D. Mitchell, Cecily A. Basquin, Sara M. Levens

**Affiliations:** Osher Center for Integrative Medicine, University of California, San Francisco, San Francisco, CA; University of North Carolina at Charlotte, Charlotte, NC; University of North Carolina at Charlotte, Charlotte, NC; University of North Carolina at Charlotte, Charlotte, NC; Department of Psychological Science, Health Psychology Ph.D. Program, University of North Carolina at Charlotte, 9201 University City Blvd., 4033 Colvard, Charlotte, NC, 28223, USA.

**Keywords:** Childhood obesity, child diet, parental emotion regulation, reappraisal, parenting self-efficacy

## Abstract

Child weight issues can be emotionally challenging for parents. The purpose of this study was to examine how parents’ ability to manage negative emotions may facilitate parenting self-efficacy and healthy parenting behaviors (e.g., providing healthy food for a child). In this study, parents (*N* = 159) of a 6–12-year-old child completed a health-specific parenting self-efficacy questionnaire and retrospectively reported their child’s daily servings of fruits and vegetables and sweets and soda. They also completed a parental emotion regulation task where they viewed film clips of families struggling with child weight challenges. During this task, parents managed their emotions by either positively reframing the situation to feel *less negative* (down-regulate negative emotions) or negatively reframing the situation to feel *more negative* (up-regulate negative emotions). We tested a mediation model examining the relations among parent emotion regulation, parenting self-efficacy, and child dietary habits. Results revealed that parents’ ability to down-regulate negative emotions was associated with lower parenting self-efficacy, which in turn was associated with greater sweets and soda consumption among children. In contrast, parents’ ability to up-regulate negative emotions was directly associated with lower sweets and soda consumption, regardless of parenting self-efficacy. Our findings have implications for healthcare practice and child weight interventions as they underscore the importance of helping parents consider the consequences of childhood obesity to encourage healthy eating behavior in families.

Childhood obesity is a serious public health concern. In 2012, over 33% of children aged 6–11 years old were overweight or obese in the United States (Ogden et al., 2014). Pediatric obesity and health behavior change are multifaceted phenomena (Ebbeling, Pawlak, & Ludwig, 2002), and accumulating evidence from both observational and experimental research suggests that the family environment plays a major role in shaping weight-related habits and obesity risk among children (Anzman, Rollins, & Birch, 2010). Child dietary habits (e.g., the consumption of fruits, vegetables, sweets, and soda) are largely influenced by parents and represent an important modifiable factor that families can target to help address or prevent unhealthy weight gain among children (Lindsay et al., 2006).

A balanced diet and proper nutrition help maintain healthy weight and decrease risk for childhood obesity (Hoelscher et al., 2013; Kretchmer et al., 1996). The nutrients found in fruits and vegetables (e.g., omega-3 fatty acids, antioxidants, magnesium, and vitamins E, C, and B) support cognitive and metabolic functioning (Kretchmer et al., 1996, Slavin & Lloyd, 2012). However, only 21% of children consume the recommended daily servings of fruits and vegetables (Cook & Friday, 2005) and 40% of calories children consume are “empty calories” from solid fats and added sugars (Reedy & Krebs-Smith, 2010). Sugar consumption in childhood is associated with weight gain (Ludwig, Peterson, & Gortmaker, 2001; Malik, Schulze, & Hu, 2006), and risk for behavioral problems (Cormier & Elder, 2007). Therefore, a combination of increased fruit and vegetable consumption and decreased sweets and soda consumption is necessary for health and wellbeing.

Parents are critical in prevention and intervention efforts to manage childhood weight (e.g., Epstein et al., 1994; Moore, Wilkie, & Desrochers, 2016), in part because they are responsible for creating a home environment that fosters healthful eating (Maitland et al., 2013). School age children (6–12 years old) who are encouraged to eat fruits and vegetables by their parents will likely eat more of such foods on their own as they enter adolescence and adulthood (Hill, 2002). Thus, parent decisions can have a meaningful impact on child dietary habits. However, little is known about the ways in which parents’ motivational processes (i.e., emotional experiences and beliefs) support or interfere with these decisions.

Weight and health challenges can give rise to a range of negative emotions for parents and families (Barlow & Dietz, 1998; Ditto, Jemmott, & Darley, 1988; Rhee, 2008; Rippe, Crossley, & Ringer, 1998). The management of these emotions through emotion regulation (i.e., a goal-driven process where one alters the intensity, occurrence, or duration of an emotion) may influence parents’ initiation and maintenance of healthy eating habits at home (Gross, 1998). One potentially beneficial emotion regulation strategy is reappraisal, which involves reinterpreting the initial appraisal of a stressful event to alter its emotional impact (Gross & John, 2003; Ray et al., 2010). Reappraisal may provide parents with the cognitive resources, motivation, and confidence needed to successfully encourage healthy dietary habits for their child. Research examining the effects of experimentally inducing negative emotion in the laboratory and training participants in reappraisal suggests this emotion regulation strategy promotes weight management behaviors, such as decreased caloric intake (Svaldi et al., 2014), as well as reduced food cravings and inhibited neural activation in response to food (Svaldi et al., 2015). Thus, parents who use reappraisal to regulate their emotions in the context of weight-related challenges may be better equipped to encourage fruit and vegetable consumption and discourage sugar consumption within the family.

Critically, reappraisal can be employed to increase or decrease negative emotions, depending on h ow the strategy is framed (Sheppes et al., 2014). For example, reappraisal framed around the positive outcomes of a situation can help a parent decrease, or down-regulate, the emotions surrounding issues of weight and health (Troy et al., 2010). Parents who feel distressed and use reappraisal to down-regulate negative emotion may be able to remain calm, focus more effectively on family eating habits, and follow through with their goals for encouraging children to make healthier food choices (Tyson, Linnenbrink-Garcia, & Hill, 2009). Alternatively, by considering the negative consequences of a situation, reappraisal can be employed to increase, or up -regulate, the negative emotions surrounding weight challenges (Ochsner et al., 2004). When parents up -regulate negative emotions, they may have an easier time identifying important negative consequences of an unhealthy child diet (Pennebaker, 1995) and experience greater awareness and control over their thoughts and actions (Moser, Most, & Simons, 2010). Research suggests that both up- and down-regulating negative emotions may support overall health and promote adaptive coping in adults (Sagui & Levens, 2016; Tugade, Fredrickson, & Barrett, 2004). Thus, these two strategies may differentially influence parents’ encouragement of healthy child dietary habits.

Effective emotion regulation may also affect health decision making indirectly, by facilitating other psychological processes, such as health-focused parenting self-efficacy—parents’ confidence that their actions will result in desired health outcomes for their child (Dumka et al., 1996; Healey et al., 2018; Hess, Teti, & Hussey-Gardner, 2004). Self-efficacy is important for the initiation and maintenance of behavior change, and parenting self-efficacy has been associated with greater satisfaction with parenting (Coleman & Karraker, 2000), active coping styles (Dumka et al., 1996), and the use of parenting practices that promote child achievement (Elder et al., 1995).

Parents who feel more efficacious regarding their ability to implement healthful behaviors at home are more likely to have children who eat healthy diets. For example, one study found that parents with greater health -focused parenting self-efficacy had school-age children who consumed greater amounts of fruits and vegetables and exercised more (Ice, Neal, & Cottrell, 2014). Other work has shown that parents reporting lower health-specific self-efficacy had children with higher body mass indexes (Ekim, 2016).

Emotion regulation may be critical in facilitating parenting self-efficacy and subsequent parenting behaviors. Regulating emotions is a goal-driven process whereby an emotion goal is activated (e.g., to increase or decrease an emotion), and the individual uses an emotion regulation strategy (e.g., reappraisal) to pursue that goal (Tamir et al., 2019). Emotion regulation is a form of self-regulation because it is a skill involving the control of oneself in the pursuit of longer-term goals, whereas self-efficacy is the belief in one’s innate ability to achieve her/his goals (Bandura, 1982). Self-regulation may occur explicitly or implicitly (Gyurak, Gross, & Etkin, 2011), while self-efficacy is a conscious belief that requires an explicit subjective evaluation. The explicit and implicit regulatory processes of emotional control may therefore underlie more conscious beliefs in parenting efficacy: when a parent is more skilled in controlling their emotions, they are more likely to feel efficacious in their parenting behaviors. In fact, a recent study testing the directionality of self-regulation and self-efficacy associations in the context of weight management behaviors found that self-regulation was a stronger predictor of change in self-efficacy than vice versa. (Annesi & Vaughn, 2017).

Further, children’s dietary habits can be difficult to change and may be characterized by frustration, disappointment, and setbacks (Limbers, Turner, & Varni, 2008), which require self-regulation for goal persistence. Parents who are more skilled at reappraising emotions may be better equipped to flexibly cope with dips in motivation that may come with efforts to manage their child’s dietary habits. These parents may feel more confident in their parenting role and their ability to foster healthy behavior among their children, even when they encounter difficult emotions (Bandura, 1997). Evidence suggests that parental emotion and self-regulation influences childhood health outcomes (Healey et al., 2018) and is associated with greater self-efficacy beliefs (Bandura & Cervone, 1986; Bandura et al., 2003). Reappraisal may therefore encourage greater health-specific parenting self-efficacy by offering greater control over emotions, thoughts, and behavioral intentions in ways that motivate parents and foster confidence in healthful parenting (Healey et al., 2018). Additionally, the different motivational mechanisms associated with up- or down-regulating negative emotions using reappraisal may affect whether a parent feels more confident in increasing healthy food intake or decreasing unhealthy food intake. Thus, because parenting self-efficacy is the belief that one can achieve parenting goals, emotion regulation that occurs in the context of goal-setting (e.g., parental regulation in a family context) should support goal success and thereby self-efficacy.

Despite the potential importance of parental emotion regulation and self-efficacy for managing child dietary habits, these factors have not been examined together and are not a focus of treatment programs for child weight (Epstein et al., 1994; Golan, Fainaru, & Weizman, 1998; Kitzman-Ulrich et al., 2010). Lack of attention to the way parents manage their emotions in response to child weight challenges may be an impediment to supporting healthy behavior among families. As a first step to address this, we tested a theoretical model that investigates the following research questions: 1) what is the direct effect of parents’ ability to implement reappraisal to up- and down-regulate negative emotions on child consumption of both fruits/vegetables and sweets/soda? And 2) what is the possible mediating role of health-focused parenting self-efficacy in this association? Due to the limited prior work in this area, we consider our research model to be exploratory and present research questions as opposed to specific hypotheses. The goal of our study is to better understand parents’ emotional and motivational processes as they relate to child diet, and we hope this work might inform parent- and family-focused weight interventions for children.

## Method

### Participants

We recruited a sample of 249 participants who reported being the parent of a child between 6 and 12 years old via Amazon’s Mechanical Turk (MTurk), a participant recruitment tool for online research. MTurk studies have been found to recruit a diverse set of respondents that represent the national population in comparison to college samples or other Internet populations (Paolacci & Chandler, 2014). MTurk registrants needed to be 18 years or older, fluent in English, live in the United States, and be a parent to one or more children aged 6 to 12 years old to participate. To be conservative, we applied strict data quality tests that excluded 90 participants because they did not pass the emotion regulation task attention checks (*n* = 35; see Measures section) or their video timing indicated that they did not watch the full video (*n* = 55) (see Data Analysis Plan section for more detail). The reported decrease in sample size due to quality control measures is common for behavioral MTurk studies with a similar design (Sagui & Levens, 2016). The final sample size included in analyses was 159 participants. Reported data patterns were similar when the full sample was included (see Supplementary Material for results in the full sample).

On average, participants were 34.55 ± 6.16 years old (range: 22–50 years old) and 74.8% identified as female. The participants represented the following self-identified racial/ethnic groups: 74.8% non-Hispanic white, 9.4% non-Hispanic African American, 8.8% Hispanic white, 5% non-Hispanic Asian American, and 1.9% bi-racial, multi-racial, or ‘other’. Regarding income, respondents represented the following self-identified pre-tax household income categories: 2.5% reported <$10,000, 10.2% reported $10,000-$24,999, 34.4% reported $25,000–49,999, 27.4% reported $50,000-$74,999, 19.1% reported $75,000 to $99,999, and 6.4% reported >$100,000. Parent body mass index (BMI) was, on average, 27.22 kg/m^2^ (*SD* = 6.57 kg/m^2^) and the average child BMI was at the 52^nd^ percentile (*SD* = 34.07).

### Procedure

Upon selecting the study on MTurk, participants were provided a link to the survey hosted on Qualtrics survey software. Informed consent was obtained from all individual participants included in the study. They were asked to complete the study in a quiet room free from distractions. At the beginning of the study, we asked respondents to answer several demographic questions about their child, including the child’s age, gender, height, weight, grade, and favorite activities. Participants then completed a set of randomized questionnaires, including parenting self-efficacy, child diet, and social desirability. Then, they completed the Parental Emotion Regulation Ability (PERA) task where we measured their ability to up- and down-regulate negative emotions using reappraisal. Finally, participants answered demographic questions about themselves. The entire session lasted approximately 1.5 hours. Participants were compensated with $5.00, a rate consistent with other behavioral MTurk studies of similar length and difficulty at the time this research was conducted. This study was approved by the university Institutional Review Board.

### Measures

#### Parental emotion regulation ability.

**Figure 1.**
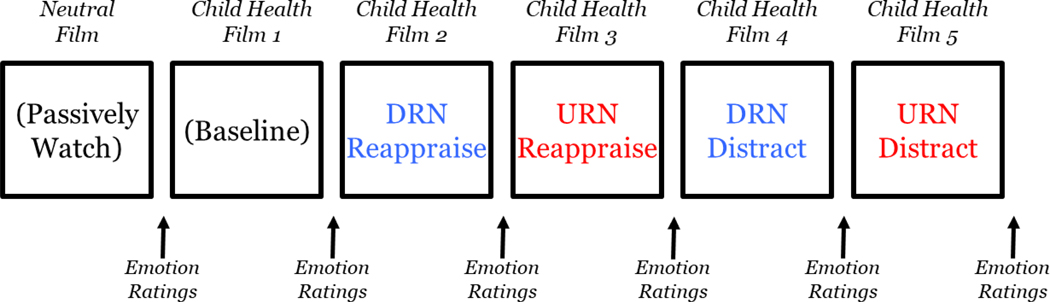
Schematic of experimental procedure for the Parental Emotion Regulation Ability (PERA) task. DRN = Down-Regulate Negative, URN = Up-Regulate Negative. The order of instructions for which strategy to implement was randomized across participants. Following each film clip, participants rated the greatest amount of emotion experienced to 11 emotion prompts (positive emotions: amusement, happiness, joy, love; negative emotions: anger, anxiety, disgust, fear, guilt, sadness, shame) on a nine-point Likert scale (1 = *Not at all*, 5 = *Somewhat*, 9 = *Extremely*).

Parents’ ability to up- and down-regulate negative emotions using reappraisal was assessed with a novel Parental Emotion Regulation Ability (PERA) task that is based on an existing reappraisal ability task (Troy et al., 2010). The PERA task is a behavioral task that can assess parents’ ability to change their own emotions toward emotion-inducing stimuli. Briefly, participants were shown pre-tested emotion-eliciting films and were instructed to use different emotion regulation strategies to change their emotions (see Figure 1 for the task schematic). Delta method change scores using post-film emotion ratings were used to determine participants’ ability to regulate their emotions based on the instructed strategy.

To develop the PERA task, we used standard procedures suggested by Rottenberg, Ray, and Gross (2007) for developing an emotion-eliciting paradigm using films. First, we selected ten film clips that met inclusion criteria (approximately 150s in length, elicited a moderate amount of negative emotions, and depict ed a child-focused obesity-related health situation). We conducted pilot testing of the stimuli in a separate MTurk sample of 113 parents of children between 6 to 12 years old (*M age* = 34.77 years, *SD age* = 7.34 years, 58.4% female; 75.2% non-Hispanic white). Respondents watched each video, provided ratings of their positive and negative emotions toward the stimuli, and answered open-ended qualitative questions about their appraisals of the stimuli. We averaged the emotion ratings to create a negative composite and positive composite score for each film and qualitatively coded each appraisal statement. We selected five film clips that had the highest negative composite ratings, elicited more negative than positive emotion, and had comparable potential to be positively and negatively reappraised. The final stimuli ranged in length from 150s to 200s and featured a story of a parent or family with an “obese” child. The stimuli all utilize a documentary film style to follow a family’s experience and challenges with the child’s weight and health behaviors (see Supplementary Material for links to the film clips).

In the PERA task, parents first viewed one neutral film (used in Troy et al., 2010) followed by the five child-focused, health-related film clips. To obtain a baseline negative emotion rating, participants were told to watch the first clip carefully. For the remaining four clips, participants were instructed to up- or down-regulate their negative emotions using reappraisal or distraction (the present study presents reappraisal findings only). A repeated measures design was utilized to avoid habituation or regression to the mean. Specifically, the order of the film clip presentation was the same, but the framing (up- or down-regulation) and strategy (reappraisal or distraction) instructions were counterbalanced across participants. Reappraisal instructions were based on Troy et al.’s (2010) instructions. In the down-regulation with reappraisal condition, we instructed participants to think about the situation they were viewing from a more positive perspective to decrease its emotional impact. In the up-regulation with reappraisal condition, we instructed participants to think about the situation they were viewing from a more negative perspective to increase its emotional impact (the full reappraisal instructions are provided in the Supplementary Material).

After viewing each film clip and implementing the emotion regulation strategy, participants completed a post-film questionnaire in which they were instructed to reflect on how they felt while watching the previous film clip. Participants first rated the greatest amount of 11 discrete emotions they experienced, including 4 positive emotions (amusement, happiness, joy, and love) and 7 negative emotions (anger, anxiety, disgust, fear, guilt, sadness, and shame) (Rottenberg, Ray, & Gross, 2007; Troy et al., 2010). Discrete emotions were rated on a 9-point Likert scale (1 = *Not at all*, 5 = *Somewhat*, 9 = *Extremely*). Next, an attention check question was presented about the film’s content; participants were not included in analyses if they answered this question incorrectly. The amount of time participants spent on the video page was also recorded.

A negative emotion composite score was created for each video by standardizing each emotion rating into a Z score and then averaging the seven standardized negative emotion ratings for each film clip. We calculated a total of five negative emotion composite scores for each participant (one for each child-focused health-related clips). To calculate parental emotion regulation ability, we calculated change scores that are consistent with those employed by Troy et al. (2010). To calculate parental ability to down-regulate negative emotions (DRN), the negative emotion composite ratings given after the film clip in which parents used reappraisal to decrease negativity was subtracted from the composite created from the baseline emotion-eliciting film to create a DRN change score, with higher scores indicating greater parental DRN. Similarly, parental ability to up-regulate negative emotions (URN) was calculated by subtracting the baseline negative emotion composite from the negative emotion composite created from the ratings given after the film clip in which parents used reappraisal to increase negativity, with higher scores indicating greater parental URN.

#### Parenting self-efficacy.

Parenting self-efficacy was measured using a modified version of the Parenting Self-Assessment Scale (Shepard, 2012; Shepard et al., 2012) that was adapted to be health-specific. This 15-item measure included statements such as “I know what to do to keep my child at a healthy weight,” and “I know what to do when my child makes unhealthy choices regarding food and activity.” Responses were noted on a 3-point scale (1 = *no*, 2 = *somewhat or sometimes*, 3 = *yes*), and all items were averaged if the participant responded to at least 12 of the items. Higher scores indicate greater health-specific parenting self-efficacy. The adapted scale had excellent psychometric properties in the present sample. This scale demonstrated strong internal consistency reliability (*α* = .94) and construct validity through its significant correlation (*r* = .40, *p* < .001) with another general measure of parenting self-efficacy (Parenting Sense of Competence, self-efficacy subscale; Gibaud-Wallston & Wandersman, 1978; Johnston & Mash, 1989). The items for our parenting self-efficacy measure are in the Supplementary Material.

#### Child dietary habits.

Child dietary habits were assessed through a food frequency questionnaire (FFQ) developed based on the Paffenbarger Physical Activity Questionnaire (Paffenbarger et al., 1993). FFQs are typical methods of measuring dietary intake in cross-sectional survey research (Burrows et al., 2013; Ludwig, Peterson, & Gortmaker, 2001; Serdula et al,, 2001; Yaroch et al., 2012) and have been found to reliably estimate dietary intake among children and adults (Andersen et al., 2004; Blum et al., 1999; Yaroch et al., 2012). Additionally, parent report of child dietary habits is common, and it has been suggested that school-aged children under 12-years of age cannot complete a FFQ as reliably as older children and adults (Burrows et al., 2013). Of importance, child dietary intake measured using FFQs is associated with actual child weight (Berkey et al., 2000; Ludwig, Peterson, & Gortmaker, 2001). Test-retest reliability of FFQs is strong for both adult self-report and parent reports about a child (Serdula et al., 2001; Yaroch et al., 2012). Overall, FFQs are a frequently used and reliable measure of children’s dietary intake.

##### Child fruit and vegetable consumption.

Child fruit consumption was assessed by asking parents to report the average number of servings of fruit that their child typically eats per day. Parents were told that one serving of fruit is equal to: 1 medium fruit,. ¼ cup dried fruit, ½ cup fresh, frozen, or canned fruit (United States Department of Agriculture, 2017). Similarly, to assess vegetable consumption, parents were asked how many servings of vegetables their child typically eats per day. Parents were told that one serving of vegetables is equal to: 1 cup raw leafy vegetable and ½ cup of cut-up raw or cooked vegetable (United States Department of Agriculture, 2017). Parent responses for fruit and vegetable consumption were summed to create a composite score with higher values indicating greater child fruit and vegetable consumption.

##### Child sweets and soda consumption.

Child sweets consumption was assessed with two questions. The first asked parents to report the number of servings of sweets and dessert foods their child typically eats in one day. They were given examples of one serving of sweets and dessert foods, including: ½ cup of ice cream, 3 graham cracker squares, ½ slice of cake, 1 cookie (United States Department of Agriculture, 2017). The second question asked parents to report the number of servings of soda (12 ounce can of soda) their child typically consumes in one day. Parent responses for sweets and soda consumption were summed to create a composite score with higher values on this variable indicating greater child sweets and soda consumption.

#### Covariates.

Our analyses carefully considered several potentially confounding factors known to influence emotion regulation, self-efficacy, and parent reports of child diet, including parent gender (Blissett, Meyer, & Haycraft, 2006), parent BMI (Liem, Mars, De Graaf, 2004), child BMI (West et al., 2010), household income (Jones et al., 2010), parental emotional reactivity during the emotion regulation task (Sagui & Levens, 2016; Troy et al., 2010), and social desirability (Bornstein et al., 2015). Parent gender was assessed by asking participants to indicate if they were male (0) or female (1). Parent BMI was calculated from self-reported height and weight, which has been shown to be valid (Christian et al., 2013). We calculated child BMI percentile from parent reports of the child’s height, weight, and age using the CDC BMI Percentile Calculator for Child and Teen (Centers for Disease Control and Prevention, 2019). Household income was assessed by asking participants the range that best described their pre-tax household income in the last year on a seven-point scale (1 = <$10,000, 6 = >$100,000). To ensure that DRN and URN scores were not confounded by participants’ reactivity to the films (Sagui & Levens, 2016; Troy et al., 2010), we calculated an emotional reactivity change score by subtracting negative emotion ratings to the neutral film clip from negative emotions ratings for the baseline child obesity film clip. This is a standard procedure with emotion regulation ability paradigms and higher scores on this covariate indicate more emotional reactivity from the neutral to baseline film clips. Social desirability was assessed with the Marlowe-Crowne Social Desirability Scale Short Form (Reynolds, 1982), a 13-item measure that asks about culturally approved behaviors that have a low incidence of occurring. Responses are noted in a true-false format, with more true responses for unlikely but culturally approved of behaviors (e.g., No matter who I’m talking to, I’m always a good listener), indicating greater levels of socially desirable responding.

### Data Analysis

First, the data were cleaned based on attention checks for the PERA task. Participants’ emotion ratings were removed using listwise deletion if they did not select the correct responses to each attention check question and/or did not remain on the video page for at least 75% of the neutral film and 100% of all five child-focused health-related films. We used SPSS 25.0 to clean the data and examine descriptive statistics, correlations, and t -tests among focal variables and covariates. Less than 1% of the data were missing, and Little’s Missing Completely at Random Test (Little, 1988) demonstrated that the data were missing completely at random, χ2 = 12.921(14), *p* = .533. Because we had a combination of continuous and censored (i.e., child diet) variables, we used weighted least squares, mean and variance adjusted (WLSMV) estimation procedures to handle missing data, as recommended by Muthén & Muthén (1998–2012).

We used MPlus 7.11 (Muthén & Muthén, 1998–2012) to conduct path modeling to examine associations among parental URN, DRN, self-efficacy, and child diet. Because each child diet variable was the sum of two count variables, we specified these outcomes as censored variables in our analyses (Muthén & Muthén, 1998–2012). As recommended by Hayes (2018), all covariates were included in analyses as predictors of our mediator (self-efficacy) and outcome (child diet) variables. We retained all covariate paths in the model regardless of statistical significance. We used the percentile bootstrap option in MPlus and specified that 10,000 bootstrap draws were to be used. To detect mediation, we examined the 95% bootstrap CIs for the indirect effects, and those not including zero indicated a statistically significant effect.

## Results

**Figure 2.**
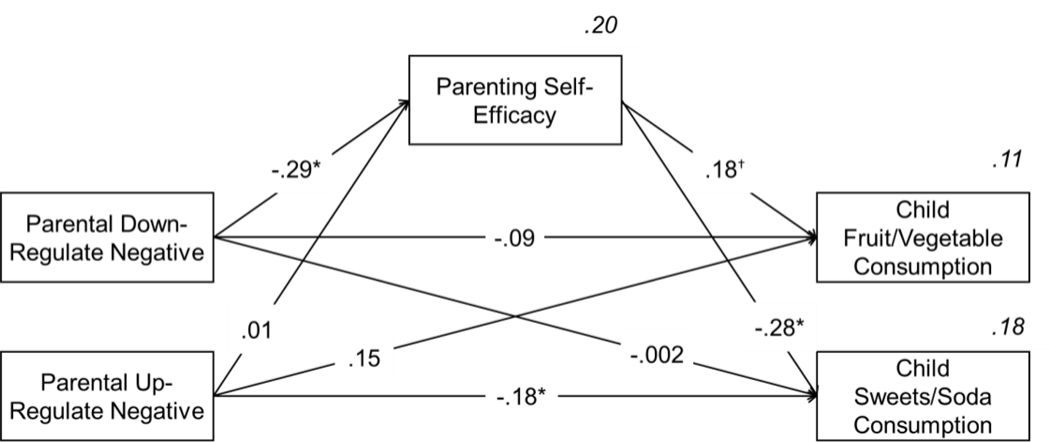
Parental emotion regulation mediation model with standardized path weights. The ability to down - and up-regulate negative emotions using reappraisal was hypothesized to associate with child fruits/vegetables consumption and child sweets/soda consumption via parenting self-efficacy. Analyses adjusted for parent gender, parent BMI, child BMI, household income, emotional reactivity, and social desirability. For clarity, covariates are not pictured. R^2^ (variance accounted for) for endogenous variables is shown in italics. *N* = 159, ^†^*p* < .10, *p < .05

Table 1 summarizes the zero-order correlations among focal study variables. Table 2 summarizes the unstandardized direct, indirect, and total effects of parental DRN and URN on parenting self-efficacy and child dietary habits. We present unstandardized effects in-text and standardized effects in Figure 2.

The final model accounted for 11% of the variance in child fruits/vegetables consumption and 18% of the variance in child sweets/soda consumption. With respect to the covariates included in the model, gender was significantly associated with parenting self-efficacy (*b* = −.157, SE = .058, *p* < .01), such that fathers reported greater parenting self-efficacy than mothers. None of the other associations between covariates and focal variables reached traditional levels of significance; however, there were several trend-level associations. Specifically, child BMI and social desirability were associated with parenting self-efficacy (child BMI: *b* = −.002, SE = .001, *p* = .078 and social desirability: *b* = .017, SE = .009, *p* = .057), parent BMI was positively associated with child fruit/vegetable consumption (*b* = .052, SE = .027, *p* = .051), and parent gender and household income were associated with child sweet/soda consumption (parent gender: *b* = −.568, SE = .321, *p* = .077 and household income: *b* = .175, SE = .101, *p* = .084).

Our path model revealed that, as hypothesized, there was a significant direct effect of parental DRN on parenting self-efficacy (*b* = −.142, SE = .046, *p* < .01), whereby parents who were better at down-regulating their negative affect while viewing child obesity health challenges also reported lower self-efficacy for child health and weight behavior. Contrary to expectations, the direct effect of parental URN on parenting self-efficacy was not significant. As predicted, results also revealed that the direct effect of parenting self-efficacy on child fruit/vegetable consumption approached significance (*b* = .912, SE = .553, *p* = .099), and the effect on sweets/soda consumption was significant (*b* = −1.102, SE = .480, *p* < .05). Parents who reported greater self-efficacy for child health and weight behaviors also reported their child consumed more fruits and vegetables and less sweets and soda. Finally, the direct effects of parental DRN and URN on child fruits/vegetables consumption were not significant; however, the effect of parental URN on child sweets/soda consumption was significant (*b* = −.345, SE = .170, *p* < .05). Parents who were better at up-regulating their negative affect in the context of viewing child obesity health challenges, also reported that their child consumed less sweets and soda.

As expected, our findings also demonstrated a significant indirect effect of parental DRN on child sweets/soda consumption through parenting self-efficacy, 95% bootstrapped CI [.013, .349]. Regarding the total effects, parental DRN did not have a significant total effect on either child diet outcome, and the total effect of parental URN on sweets/soda consumption approached significance (90% bootstrapped CI [−.665, −.049]) but did not reach traditional levels of significance, as indicated by the 95% bootstrapped CI [−.744, .009]. .

## Discussion

The present study investigated how parental emotion regulation ability related to child dietary habits and whether parenting self-efficacy acted as a mediator in this process. Prior research suggests that emotion regulation may influence behavioral responses to stressful situations (Lazarus, 1999; Troy et al., 2010); thus, a parent’s ability to increase and decrease negative emotions in the context of child health challenges may be an important process to consider when trying to understand parents’ encouragement of their children’s healthy eating behavior. A strength of this study is that we examined parents’ ability to both *increase* and *decrease* negative emotions, which allowed us to shed light on how each process impacts children’s dietary habits.

Our first research question tested the direct associations between parents’ emotion regulation abilities and child diet. We found several relationships among our variables that constituted small to medium effect sizes. Specifically, we found that, although parents’ down-regulation (DRN) ability was associated with less child fruits/vegetable and more sweets/soda consumption in correlation analyses, these associations were attenuated in the path analysis when controlling for the ability to up-regulate negative emotions (URN). In contrast, parents’ URN skill was associated with more child fruits/vegetable and less sweets/soda consumption in correlation analyses, and the association with sweets/soda remained significant in the path analysis.

Negatively-valanced emotions, such as fear and worry, are elicited in response to perceived, personally-relevant threats (Easterling & Leventhal, 1989). Therefore, when parents are more attuned to the harmful consequences of unhealthy weight gain that can arise for their children, they may be more apt to increase their negative emotions in response to child health and weight challenges . This process may motivate parents to support healthy family behaviors (e.g., restricting the availability of sweets and soda at home) and to set clear expectations regarding child diet (e.g., family rules regarding healthy snacking and mealtime options). These findings align with the Protection Motivation Theory, which suggests that appraisals of the severity of a threat (e.g., obese child) and responses to that threat (e.g., reappraisal and self-efficacy) lead to goal intention and subsequent behavior (Rogers, 1975). Therefore, parents that have a greater capacity to URN may perceive obesity and their child’s susceptibility to it as a serious threat, motivating them to reduce their child’s sweets and soda intake.

In the correlation and path analyses, greater health-specific parenting self-efficacy was associated with more child fruit and vegetable consumption and less sweets and soda consumption. This is consistent with past research demonstrating the influence of parenting self-efficacy on a range of childhood behaviors including dietary habits (Ice, Neal, & Cottrell, 2014), physical activity (Ice et al., 2014; Smith et al., 2010), and mealtime eating behaviors (Heerman et al., 2017). Our results extend this work to suggest that parents’ confidence about their ability to help their child maintain a healthy weight has beneficial associations with the foods their child typically eats. Importantly, reappraisal ability was controlled for when testing the association between parenting self-efficacy and child diet. This indicates that health-focused self-efficacy shares a significant relation with child diet independent of reappraisal ability, although this confidence may be influenced by emotion regulatory skill.

Thus, our second research question tested whether parenting self-efficacy served as a mechanism through which parental reappraisal ability influences child behavior. Although parents’ DRN ability had a small, but non-significant direct effect on child fruit and vegetable consumption, we found evidence of a significant indirect effect on sweets/soda consumption through parenting self-efficacy. Specifically, parents who were more skilled at decreasing their negative emotions in the context of child weight and health challenges had significantly *lower* health-focused parenting self-efficacy, and in turn reported that their children consumed *more* sweets/soda. This finding suggests that parents’ ability to decrease their negative emotional states when faced with child obesity problems may impede their ability to promote healthy dietary habits. Perhaps parents who are more skilled at focusing on the positive aspects of child weight and health challenges are less motivated to make behavioral changes. Future research should explore the possibility that viewing child health situations as uncontrollable leads to hedonic (i.e., short-term) regulatory goals and greater DRN ability, while viewing situations as controllable leads to instrumental (i.e., longer-term) regulatory goals and greater URN ability (e.g., Sheppes et al., 2014).

Although a greater ability to DRN appears to have detrimental effects on child sweets/soda consumption via its association with parenting self-efficacy, a greater ability to URN seems to have beneficial effects on child diet regardless of self-efficacy. Our results suggest that increasing negative emotions in this child health context may encourage parents to initiate action to avoid potential harmful effects to their child’s health, such as limiting sweets and soda consumption. Increasing negative emotions is theorized to increase motivation and desire for successful outcomes (Schmader & Lickel, 2006), as well as enact preventive strategies to reduce the likelihood of potential negative outcomes (Nabi, 2003; Pennebaker, 1995). In other words, parents who have a greater ability to URN may be more motivated to encourage healthful eating and not rely on self-efficacy; perhaps the salience of the negative health outcomes is sufficient to directly influence behavior.

Applying classic negative reinforcement learning (Domjan, 2015) to this health context, negative emotions about one’s weight signal the need for behavioral change to remove the negative stimulus (excessive weight). Accordingly, URN ability may facilitate healthful behavior change by increasing the motivation to eat healthier to reduce obesity risk. Critically, although emotion regulation literature purports that reducing negative emotions in negative situations may be adaptive and beneficial (Gross & John, 2003; Tugade et al., 2004), our results suggest that in the context of health-specific parenting behaviors, parents who decrease their negative emotional state may be less likely to encourage healthy food consumption. Future work is needed to clarify why DRN ability in this context may be problematic, including investigating whether it facilitates decreased motivation for behavioral change; this, in turn may reduce concern regarding the negative impacts of unhealthy child diet and weight and/or increase maladaptive use of food to regulate negative emotion (Blissett, Haycraft, & Farrow, 2010).

Importantly, we do not believe these findings promote parents’ use of weight stigmatization. The emotion regulation instructions for the URN condition provided examples of how to engage in negative reappraisal and were explicit in asking participants to consider the potential negative consequences of the film. A person’s skill at engaging in reappraisal to evaluate the future negative impact of a situation is important for learning and motivation and is a different process than encouraging weight stigmatizing appraisals. For example, it is possible that parents may worry that their child could be s tigmatized if they become obese—as this could be a negative consequence—but that does not mean that they will enact the stigmatizing. Further, the thoughts parents use to URN and activate behavior change are starting points for making healthy changes in the family. These reappraisals do not imply something is inherently wrong with the child, and the appraisals do not need to be shared with the child for the parent to adjust their own feeding behavior. Thus, we believe that the process of increasing negative emotions in this context is unique and does not reflect weight stigma or shaming.

Instead, we believe that processing negative emotions through reappraisal allows for further elaboration of thoughts and future-oriented thinking towards potential negative outcomes (Goldin et al., 2012). As a result, increased negative emotions through reappraisal may encourage the development of behavior intentions and identification of positive outcome expectancies, for example, “If I feed my child fruits and vegetables, I will reduce my child’s risk for obesity”. However, in the context of decreasing negative emotions, a parent may not develop behavioral intentions that drive healthful behavior, resulting in no outcome expectancies and little attention directed to the amount of healthy or unhealthy food their child can access.

Our results contribute to the literature by providing a more comprehensive view of the way parental emotion regulation ability influences child eating behaviors. Accordingly, the present study had several strengths, including a community sample, the use of a novel behavioral task, assessing both DRN and URN via reappraisal, and the assessment of healthy and unhealthy child dietary habits. Further, all our measures were specific to parenting and health, including the PERA task and the parenting self-efficacy questionnaire. Assessing these constructs in a health context allowed for the examination of these processes in a more focused manner.

### Limitations and Future Research

Despite these strengths, our findings should be generalized with caution. As a preliminary investigation of these topics, the present study employed cross-sectional tests of mediation which may be biased and cannot establish directionality (Maxwell & Cole, 2007). Future research should examine these processes longitudinally to test causal pathways. This study was also conducted entirely online. Although we included strict attention checks and questions aimed at verifying a respondent’s parental status, the nature of online surveys makes it difficult to ensure participant attention and identity. Using MTurk allowed us to reach a national community sample that, although was drawn from a limited pool of MTurk users, may be more representative than typical convenience samples drawn from a single region (Buhrmester et al., 2011). This study should be replicated with an in-person community sample.

In the PERA task we assess parents’ ability to up- and down-regulate negative emotions during a distressing health-related task, not parents’ natural tendency to use these regulation strategies. Future research should assess the relation between parental regulation ability and use in a child health context. The task and analyses were designed to target distressing parent-child weight management situations in which parental resolve was being tested. Emotion regulation ability may function differently in more positive situations when parents’ resolve is not being tested (e.g., a doctor announcing that a child has lost weight since the last physical). Thus, our findings may not generalize to positive contexts where up-regulating negative emotions may be maladaptive and undermine a child’s success. Relatedly, the ability to regulate negative emotions that arise because of perceived shortcomings in others (e.g., anger) may impact parenting self-efficacy and child diet differently than the ability to regulate self-focused negative emotions (e.g., guilt). Although our goal was to combine across emotions to derive a negative affect profile, future research should test these associations with discrete negative emotions rather than a single negative emotion factor. Another limitation of our analyses was our use of delta method change scores to assess URN and DRN ability, which may not detect reliable, individual differences in true change and present a potential source of measurement error and bias (Rogosa & Willett, 1983). Further, all data were obtained from the parent, including typical child diet, necessitating future work using a multi-method or multi-rater approach to measure child diet. Nevertheless, taken together, our results highlight an interesting and novel pathway between parental emotion regulation and healthy child dietary habits and may inform efforts aimed at improving child health.

All procedures performed in studies involving human participants were in accordance with the ethical standards of the University of North Carolina at Charlotte Institutional Review Board and with the 1964 Helsinki declaration and its later amendments or comparable ethical standards.

## Supplementary Material

10826_2020_1745_MOESM1_ESM

## Figures and Tables

**Table 1:** Descriptive Statistics and Zero-order Correlations Among Focal Study Variables

Variable	*M*	*SD*	1	2	3	4	5
1. Parental DRN	.55	1.38	--				
2. Parental URN	.47	1.46	−.43^c^	--			
3. Parenting Self-Efficacy	2.64	.40	−.27^b^	.11	--		
4. Child F/V Consumption	4.53	2.03	−.14^†^	.16^a^	.22^b^	--	
5. Child S/S Consumption	2.22	1.56	.22^b^	−.24^b^	−.30^c^	−.04	--

*Note. N* = 159;

† *p* < .10,

a *p* < .05,

b *p* < .01,

c *p* < .001. DRN = Down-regulate negative, URN = Up-regulate negative, F/V = Fruit and vegetable, S/S = Sweets and soda.

**Table 2:** Summary of Effects (Unstandardized Units) for the Parental Emotion Regulation Mediation Model

	Outcome Variables							

Predictor Variables	Parenting Self-Efficacy		Child F/V Consumption			Child S/S Consumption		
	*b*	SE	*b*	SE	95%	*b*	SE	95%
					Bootstrapped CI			Bootstrapped CI
Parental DRN								
Direct effect	−.14^b^	.05	−.21	.25	--	−.004	.18	--
Indirect effect	--	--	−.13	.09	−.32, .03	.16^a^	.09	.01, .35
Total effect	−.14^b^	.05	−.34	.25	−.80, .17	.15	.20	−.26, .53
Parental URN								
Direct effect	.003	.06	.36	.22	--	−.35^a^	.17	--
Indirect effect	--	--	.003	.06	−.15, .12	−.004	.07	−.19, .11
Total effect			.37	.22	−.08, .79	−.35^†^	.19	−.74, .01
Parenting Self-Efficacy								
Direct/Total effect	--	--	.91^†^	.55	−.22, 1.97	−1.10^a^	.48	−2.02, −.16

*Note. N* = 159;

† *p* < .10,

a *p* < .05,

b *p* < .01,

c *p* < .001. All results come from a single path analysis model conducted in Mplus. *b* = Unstandardized partial regression coefficient, SE = Standard error, CI = Confidence interval, DRN = Down-regulate negative, URN = Up-regulate negative, F/V = Fruit and vegetable, S/S = Sweets and soda. Analyses adjusted for parent gender, parent BMI, child BMI, household income, emotional reactivity, and social desirability. Indirect and total effects were calculated using percentile bootstrapping (based on 10,000 bootstrapped samples).

## Highlights:

- Parents’ health-related emotion regulation influences self-efficacy and child diet
- Increases in parents’ negative health-related emotions predict healthier child diet
- Parents’ ability to manage health-related emotions predicts parenting self-efficacy
- Greater parenting self-efficacy is associated with healthier child diet
