# Supplementary material for: The Effects of Parental Emotion Regulation Ability on Parenting Self-Efficacy and Child Diet
Source: J Child Fam Stud. Author manuscript; Available in PMC 2024 Aug 29. (PMC11361363; doi:10.1007/s10826-020-01745-x)
Supplement: 10826_2020_1745_MOESM1_ESM [file NIHMS1606879-supplement-10826_2020_1745_MOESM1_ESM.docx]

**Supplementary Material**

**Results of the Mediation Models in the Full Sample**

When the 90 participants who did not pass the attention checks were included in the analyses, the reported data patterns did not change from the reported analytic sample (*N* = 159). The final model accounted for 9% of the variance in child fruits/vegetables consumption and 13% of the variance in child sweets/soda consumption. Results revealed that there was a significant, direct effect of parental DRN on parenting self-efficacy (*b* = -.11, SE = .04, *p* < .01). The direct effect of parental URN on parenting self-efficacy was not significant (*b* = .01, SE = .05, *p* = .80). Results also revealed that the direct effect of parenting self-efficacy was significant for sweets/soda consumption (*b* = -1.04, SE = .36, *p* < .01), but did not reach traditional levels of statistical significance for child fruits/vegetables, though directionality was the same (*b* = .73, SE = .41, *p* = .08). The direct effects of parental DRN (*b* = -.29, SE = .19, *p* = .14) and URN (*b* = .21, SE = .18, *p* = .24) on child fruits/vegetables consumption were not statistically significant. Further, the effect of parental DRN on sweets/soda consumption was not statistically significant (*b* = .17, SE = .15, *p* = .28). However, the effect of parental URN on child sweets/soda consumption was significant (*b* = -.34, SE = .15, *p* < .05). There was a significant indirect effect of parental DRN on child sweets/soda consumption through parenting self-efficacy, 95% bootstrapped CI [.016, .250]. Further, the indirect effect of parental DRN on child fruits/vegetables consumption reached statistical significance at the 90% bootstrapped CI [-.175, -.001] but not the 95% bootstrapped CI [-.199, .012].

**Links to Parental Emotion Regulation Ability Task Stimuli**

Neutral Film: <https://vimeo.com/110102053>

Child Health Film 1 (Baseline): <https://vimeo.com/172326909>

Child Health Film 2: <https://vimeo.com/172327048>

Child Health Film 3: <https://vimeo.com/172327101>

Child Health Film 4: <https://vimeo.com/172327821>

Child Health Film 5: <https://vimeo.com/172452848/8ff1e331c8>

**PERA Task Emotion Regulation Instructions**

Down-Regulate Negative Reappraisal Instructions

Please watch the following film clip carefully.

This time, as you watch, try to ***think* *about the situation* you see in a more positive light**. You can achieve this in several different ways.

- For example, try to imagine advice that you could give to yourself or the characters in the film clip to make yourself or them feel better. This could be advice that would help you or the characters think about the positive bearing this event could have on their lives.
- Or, think about the good things you or the characters might learn from this experience. Keep in mind that even though a situation may be painful in the moment, in the long run, it could make one’s life better, or have unexpected good outcomes.

In other words, try to **think about the situation in as positive terms as you possibly can**. This can be difficult at times, so it is very important that you try your best. It is very important that you carefully watch the film clip but think about it from a positive perspective.

Up-Regulate Negative Reappraisal Instructions

Please watch the following film clip carefully.

This time, as you watch, try to ***think about the situation* you see in a more negative light**. You can achieve this in several different ways.

- For example, try to imagine advice that you could give to yourself or the characters in the film clip to make you or them take the message of the film more seriously. This could be advice that would help you or the characters think about the possible negative impact this event could have on one's life.
- Or, think about “hard lessons” that could be learned from this experience. Keep in mind that even though a situation may be painful in the moment, the situation could become even worse with unexpected negative outcomes, and it is important to think about these potential negative outcomes so that you can be prepared.

In other words, try to **think about the situation in as negative terms as you possibly can**. This can be difficult at times, so it is very important that you try your best. It is very important that you carefully watch the film clip but think about it from a negative perspective.

**Health-Specific Parenting Self-Efficacy Measure**

(Adapted from the Parenting Self-Assessment measure developed by Shepard et al., 2012)

Instructions: Please use this scale to rate whether or not you agree with each statement. For each, think specifically about parenting your child aged 6 to 12 years old.

Response options:

1=No

2=Somewhat or Sometimes

3=Yes

1. I know what to do to keep my child at a healthy weight
2. I know what to do to make sure my child feels good about his/her weight
3. I worry about the choices I make as a parent regarding my child’s weight
4. I know how to prepare my child to engage in healthy behaviors at school
5. As a parent, I know how to handle things that happen with my child
6. I know what to do to make sure my child grows up to be a healthy person
7. I have a hard time making good choices as a parent
8. I know what to do to make sure my child avoids unhealthy behaviors
9. I make a lot of mistakes as a parent when it comes to helping my child manage his/her weight
10. I know what to do to keep my child from going down an unhealthy path
11. I know what to do when my child makes unhealthy choices regarding food and activity
12. I know how to deal with power struggles between my child and me over food and activity
13. I know what to do to keep my child on the right path with his/her weight
14. I know what works for keeping my child at a healthy weight
15. I know what to do when my child breaks a rule or doesn’t listen

**Supplementary Materials References**

Shepard, S. (2012). *Parenting self-assessment*. Unpublished scale, Department of Psychiatry and Human Behavior, Brown Medical School.

Shepard, S., Armstrong, L. M., Silver, R. B., Berger, R., & Seifer, R. (2012). Embedding the family checkup and evidence-based parenting programs in Head Start to increase parent engagement and reduce conduct problems in young children. *Advances in School Mental Health Promotion, 5*, 194-207.

Rottenberg, J., Ray, R. D., & Gross, J. J. (2007). Emotion elicitation using films. In J. A. Coan & J. J. B. Allen (Eds.), *The handbook of emotion elicitation and assessment* (pp. 9-28). London: Oxford University Press.
